# Supplementary material for: A Critical View of the Application of the APEX Software (Aqueous Photochemistry of Environmentally-Occurring Xenobiotics) to Predict Photoreaction Kinetics in Surface Freshwaters
Source: Molecules. 2019 Dec 18;25(1):9. doi: 10.3390/molecules25010009 (PMC7017383; doi:10.3390/molecules25010009)
Supplement: Supplementary file 1 [file molecules-25-00009-s001.zip › 3.2.4_gcc-4.4.0.zip!/3.2.4_gcc-4.4.0/3.2.4_gcc-4.4.0/doc/octave/html/interpreter/Plotting-the-Triangulation.html]

Plotting the Triangulation - Untitled


Next: Identifying points in Triangulation,
Up: Delaunay Triangulation


---

#### 29.1.1 Plotting the Triangulation

Octave has the functions `triplot` and `trimesh` to plot the
Delaunay triangulation of a 2-dimensional set of points.

— Function File: **triplot** (tri, x, y)  
— Function File: **triplot** (tri, x, y, linespec)  
— Function File: h = **triplot** (...)  
> Plot a triangular mesh in 2D. The variable tri is the triangular
> meshing of the points `(`x`,` y`)` which is returned from
> `delaunay`. If given, the linespec determines the properties
> to use for the lines. The output argument h is the graphic handle
> to the plot.
>
>
>
> **See also:** plot, trimesh, delaunay.

— Function File: **trimesh** (tri, x, y, z)  
— Function File: h = **trimesh** (...)  
> Plot a triangular mesh in 3D. The variable tri is the triangular
> meshing of the points `(`x`,` y`)` which is returned
> from `delaunay`. The variable z is value at the point
> `(`x`,` y`)`. The output argument h is the graphic
> handle to the plot.
>
>
>
> **See also:** triplot, delaunay3.

The difference between `triplot` and `trimesh` is that the
former only plots the 2-dimensional triangulation itself, whereas the
second plots the value of some function `f (`x`,` y`)`.
An example of the use of the `triplot` function is

```
     rand ("state", 2)
     x = rand (20, 1);
     y = rand (20, 1);
     tri = delaunay (x, y);
     triplot (tri, x, y);
```

that plot the Delaunay triangulation of a set of random points in
2-dimensions.
